# Accurate preoperative staging and HER2 status prediction of gastric cancer by the deep learning system based on enhanced computed tomography

**DOI:** 10.3389/fonc.2022.950185

**Published:** 2022-11-14

**Authors:** Xiao Guan, Na Lu, Jianping Zhang

**Affiliations:** Department of General Surgery, The Second Affiliated Hospital of Nanjing Medical University, Nanjing, Jiangsu, China

**Keywords:** gastric cancer, HER2 status, deep learning, CNN, transformer

## Abstract

**Purpose:**

To construct the deep learning system (DLS) based on enhanced computed tomography (CT) images for preoperative prediction of staging and human epidermal growth factor receptor 2 (HER2) status in gastric cancer patients.

**Methods:**

The raw enhanced CT image dataset consisted of CT images of 389 patients in the retrospective cohort, The Cancer Imaging Archive (TCIA) cohort, and the prospective cohort. DLS was developed by transfer learning for tumor detection, staging, and HER2 status prediction. The pre-trained Yolov5, EfficientNet, EfficientNetV2, Vision Transformer (VIT), and Swin Transformer (SWT) were studied. The tumor detection and staging dataset consisted of 4860 enhanced CT images and annotated tumor bounding boxes. The HER2 state prediction dataset consisted of 38900 enhanced CT images.

**Results:**

The DetectionNet based on Yolov5 realized tumor detection and staging and achieved a mean Average Precision (IoU=0.5) (mAP_0.5) of 0.909 in the external validation cohort. The VIT-based PredictionNet performed optimally in HER2 status prediction with the area under the receiver operating characteristics curve (AUC) of 0.9721 and 0.9995 in the TCIA cohort and prospective cohort, respectively. DLS included DetectionNet and PredictionNet had shown excellent performance in CT image interpretation.

**Conclusion:**

This study developed the enhanced CT-based DLS to preoperatively predict the stage and HER2 status of gastric cancer patients, which will help in choosing the appropriate treatment to improve the survival of gastric cancer patients.

## Introduction

Gastric cancer is one of the most common tumors in the world and ranks fourth in cancer-related deaths (1). Many individuals with gastric cancer are already in the late stages when they are detected, due to the atypia of early symptoms (2, 3). The tumor, node, and metastasis (TNM) stage is commonly used to assist clinicians in making treatment decisions, and for patients with advanced disease, adjuvant chemotherapy is recommended as the standard preoperative treatment (4, 5). However, the use of medical imaging for preoperative staging assessment is unsatisfactory (6, 7). Surgical resection combined with adjuvant chemotherapy or chemoradiotherapy is still the main treatment for advanced gastric cancer (4). However, even with standard treatment, the prognosis of patients with advanced gastric cancer remains poor (8, 9). HER2 overexpression is an important driver of gastric carcinogenesis and is associated with poor prognosis in advanced gastric cancer (10–12). Trastuzumab combined with standard chemotherapy can significantly improve overall survival in HER2-positive patients (4, 11, 13, 14). Accurate assessment of HER2 status is crucial in the treatment of gastric cancer (15). Detecting HER2 status by immunohistochemistry (IHC) or fluorescence *in situ* hybridization (FISH) is widespread, although they are invasive and costly (16, 17).

Identifying imaging biomarkers is critical in oncology (18). Studies have shown that using medical images can capture the biology of tumors at the genetic and cellular level (19). Enhanced CT is widely used in clinical practice and is a routine imaging examination for preoperative evaluation of gastric cancer patients (20). In recent years, deep learning (DL) has gained increasing attention in the field of oncology. Deep learning has gained increasing attention in the field of oncology recently, which can extract more information from input data (21–23). Convolutional neural networks (CNNs) are the most mature deep learning algorithms and perform well in a variety of image classification tasks (24, 25). Transformers have also received extensive attention in image classification and detection (26–28). Research has confirmed that, under certain conditions, the predictive performance of DL models is not inferior to that of human experts (29, 30).

Therefore, this study aimed to develop the DLS for preoperative prediction of staging and HER2 status in patients with gastric cancer. We also built a simple web service (web) to make this prediction more accessible to clinicians. To our knowledge, this has not been reported in any published study.

## Materials and methods

**Figure 1:**
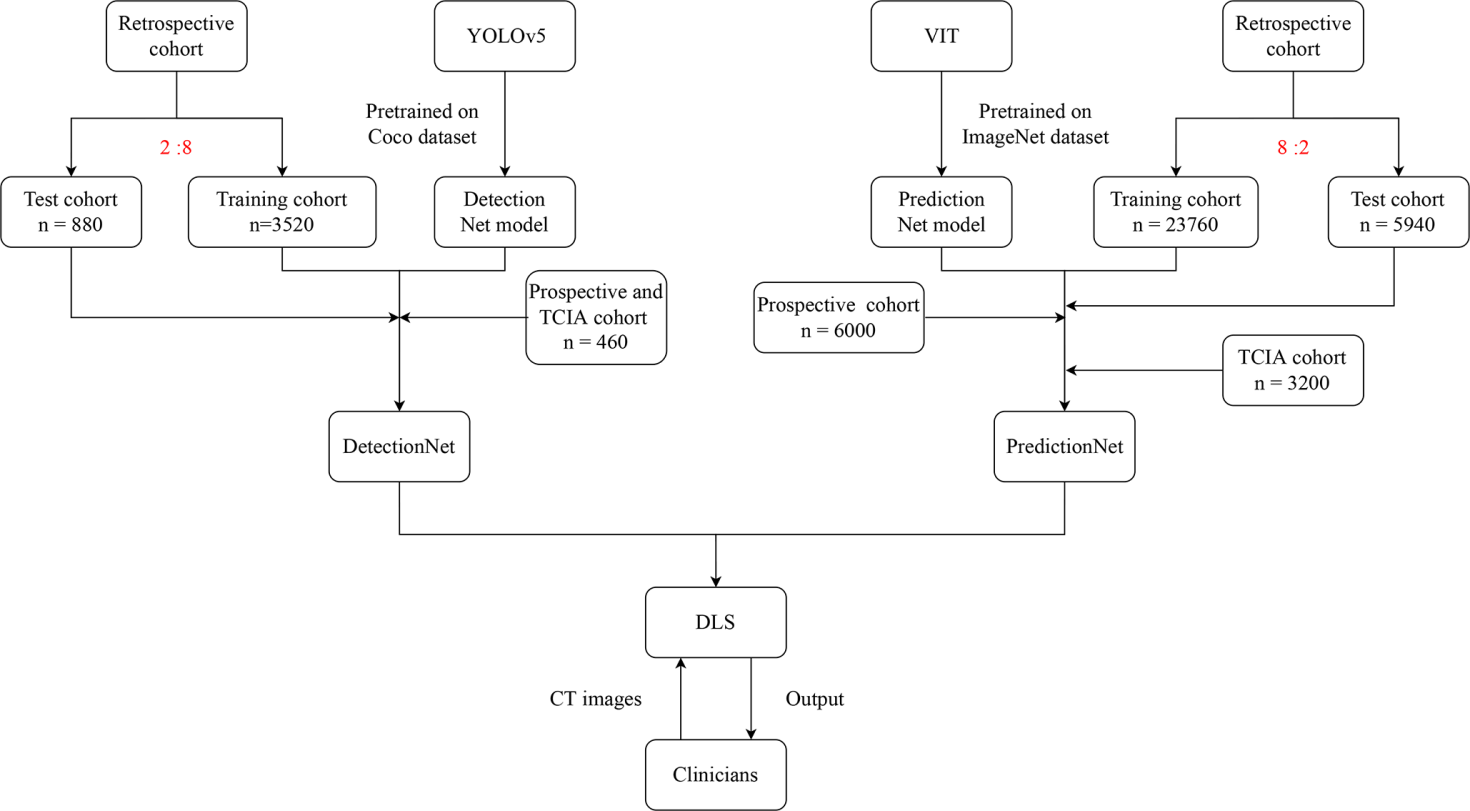
The flowchart of this study.

**Figure 1** depicted the workflow of this study.

### Patients

We collected three different cohorts. Our research team retrospectively collected clinical data from all gastric cancer patients from January 2017 to June 2021, and a total of 297 patients participated in this study. We also collected CT images and clinical information of 40 patients from the TCIA database. In addition, we prospectively and continuously collected the clinical data of 60 gastric cancer patients from October 2021 to April 2022. The inclusion criteria included enhanced CT within 1 week before gastrectomy, postoperative pathology confirmed as gastric cancer, post-gastrectomy HER2 status testing, clear HER2 status, and no preoperative chemotherapy or radiotherapy. The exclusion criteria included poorly dilated stomach or artifacts on CT images, small gastric cancer lesions that are difficult to identify, and the inability to determine HER2 status in patients with gastric cancer. **Supplementary Material** detailed the sample size assessment process (**Figure S1**) and the data collection (**Figures S2A–C**).

### CT image acquisition

CT examinations were performed on a 64 Dual Source CT. The patient was instructed to fast for more than 8 hours and to inject anisodamine 20 mg intravenously to avoid gastric motility. Besides, all patients were asked to take 1000ml of warm water orally to dilate the stomach before the examination and hold their breath during the examination. After the non-enhanced abdominal CT scan, the patients were intravenously injected with 1.5 mL/kg of iodinated contrast medium (ioversol injection 320 mg I/mL, Jiangsu Hengrui Pharmaceuticals Co.,Ltd, Jiangsu, China) at a flow rate of 3.0 mL/s by an automatic pump syringe. After the contrast agent injection starts, when the contrast agent concentration reached 100 Hu, the imaging after 8 seconds is the arterial phase, the imaging at 21 seconds after the arterial phase imaging is the venous phase, and the imaging at 90 seconds after the venous phase imaging is the delayed phase. The parameters of the CT scan were as follows: tube voltage 120 kV, tube current 150 - 300 mA, field of view 30 - 50 cm, matrix 512 × 512, rotation time 0.5 seconds, pitch 1.0, and images were reconstructed with section thicknesses of 2 mm.

### CT images collection

Studies confirmed that features extracted from the enhanced CT arterial phase images had better predictive performance than the portal venous phase (31, 32). Therefore, we resampled the enhanced CT arterial phase images. The resampled voxel sizes were set to 1×1×1 mm³ voxels to standardize the slice thickness. Two radiologists reviewed the patient’s enhanced CT arterial phase images and both of them had more than eight years of medical imaging experience. The evaluation processes of the two doctors were independent of each other, and they did not know the patient’s pathological information. For each patient’s CT images, they took a total of five images of the largest cross-section of the tumor and annotated the images, using five consecutive slices (maximum lesion). They marked the tumor location on these images. If their opinions disagreed, the opinion of another chief physician with 15 years of experience in medical imaging will be finally adopted.

### Dataset construction

We screened the five consecutive axial slices with the largest tumor area from the CT images of each patient for the construction of the dataset (33). Specifically, one of the slices was located in the largest section of the tumor, and then, with this slice as the center, the upper two slices and the lower two slices were selected, for a total of five slices.

We retrospectively collected 1100 images for automatic tumor location detection and predicted staging, including 370 stage I images, 360 stage II images, and 370 stage III images. Additional images were then obtained using cropping, flipping, and rotating. This approach reduced the possibility of overfitting when the model processes the dataset (34). A total of 4400 images were obtained. Then, we randomly divided these images into training and test cohorts in a ratio of 8:2. The training cohort was used for model training, and the test cohort was used for model validation. We also collected 160 images from the TCIA cohort and 300 images from the prospective cohort. As the vast majority of patients in the TCIA cohort were in stage III, we mixed the two cohorts as an external validation cohort to further validate the performance of the model.

We retrospectively collected a total of 1485 CT images for HER2 status prediction, including 800 HER2-negative and 685 HER2-positive images. We used image enhancement techniques to perform 19 image transformations to expand the original dataset. We use the “transforms” function for image enhancement, using three methods, including Crop, Filp and Rotation, Transform. The specific method names are as follows: RandomCrop, CenterCrop, RandomResizedCrop, RandomRotation, RandomVerFilp, RandomHorFilp, Normalize, RandomErasing, Pad, ColorJitter, RandomGrayscale, Affine, RandomOrder. We randomly split these images into a training cohort and a test cohort in a ratio of 8:2. The training cohort was used for model training, and the test cohort was used for model validation. We also collected 160 and 300 images from the TCIA cohort and the prospective cohort, respectively. After performing the same image enhancement process on them, they were used as two external validation cohort for further validation of the model. All the images were normalized.

### Model construction

We decomposed the model into two tasks. The first task was to detect the tumor location of gastric cancer in enhanced CT images and predict their stage (DetectionNet). The second task predicted the HER2 status of the tumor (PredictionNet).

**Figure 2:**
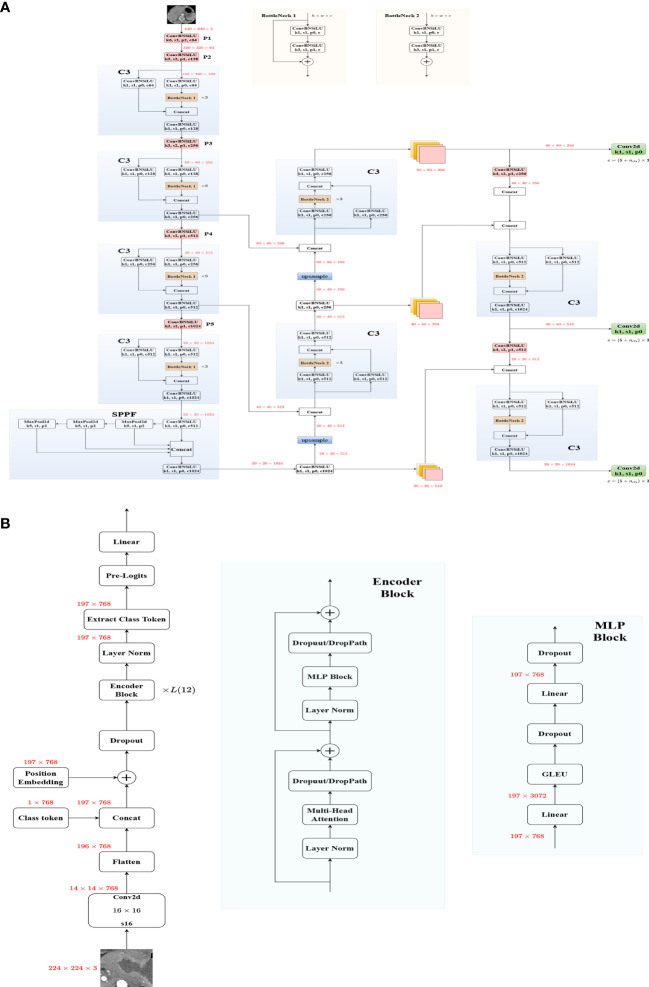
The overall network structure of Yolov5 **(A)** and VIT **(B)**.

Yolov5 was used to build the DetectionNet, which was pre-trained on the Coco dataset. Data enhancement techniques such as image translation and image scale were used in the construction of the DetectionNet. The architecture was shown in **Figure 2A** and we did not modify YOLOv5.

When building the PredictionNet, we preprocessed the images of the training cohort and the test cohort differently (35). EfficientNet is one of the most powerful CNNs, which has achieved the highest accuracy on the ImageNet top1 while requiring fewer computing resources than other models (36). Therefore, we chose EfficientNet (**Figure S3**) and EfficientNetV2 (**Figure S4**) to build the CNN models. Transformer-based image structure has strong non-local feature extraction ability, VIT (**Figure 2B**), and SWT show great performance and are considered as strong backbones (27, 28, 37). Both of them were used to build the transformer models. They were all pre-trained on the ImageNet dataset (38, 39). **Supplementary Material** detailed the training process of the model.

### Model evaluation

For DetectionNet, compute_loss was divided into three parts: cls_loss, box_loss, and obj_loss. Cls_loss is the Classes loss, which is used to calculate whether the anchor box and the corresponding calibration classification are correct (BCE loss). Box_loss is the Location loss, which is the error between the predicted box and the calibration box (CIoU loss). Obj_loss is the Objectness loss, used to calculate the confidence of the network (BCE loss). The formula of the loss function is detailed in the **Supplementary Material**. The calculation of loss was performed on each layer of feature maps. Confusion matrix, mAP, and Precision-Recall (P-R) curves were used to further evaluate the performance of the DetectionNet.

For PredictionNet, we evaluated the classification performance of the networks by accuracy and loss value and selected the best network. At the same time, the receiver operator characteristics (ROC) curves and P-R curves were also used for network evaluation. Gradient-weighted Class Activation Mapping (Grad-CAM) was used to visualize the output of a given layer in deep learning (40).

### DLS construction

The DLS consisted of two parts, including tumor detection and staging by the DetectionNet and HER2 status prediction by the PredictionNet. We visualized the DetectionNet based on “pyqt5”, and encapsulated the PredictionNet into an executable file based on”pyinstaller”. To further facilitate clinicians to use the DLS, we also built a simple web based on “flask”, which was applied to all Internet Protocol (IP) in the hospital’s local area network, and all hospital staff could use the service.

## Results

### Patient characteristics

A total of 397 patients were included in this study. **Table 1** and **Table 2** summarized the clinical findings of the retrospective, TCIA, and prospective cohorts.

**Table 1:** Patient characteristics in each cohort (DetectionNet).

Clinical characteristics		Retrospective cohort	TCIA cohort	Prospective cohort	p
Age (years), (mean ± SD^a^)		64.28 ± 10.93	65.22 ± 10.079	63.33 ± 11.613	0.719
Sex, n (%)					0.467
	Male	157 (71.4)	26 (81.3)	42 (70)	
	Female	63 (28.6)	6 (18.8)	18 (30)	
T stage, n (%)					0.000*
	T1	65 (29.5)	0 (0)	15 (25)	
	T2	34 (15.5)	1 (3.1)	17 (28.3)	
	T3	64 (29.1)	19 (59.4)	11 (18.3)	
	T4	57 (25.9)	12 (37.5)	17 (28.3)	
N stage, n (%)					0.000*
	N0	88 (40)	6 (18.8)	18 (30)	
	N1	30 (13.6)	5 (15.6)	15 (25)	
	N2	13 (5.9)	11 (34.4)	12 (20)	
	N3	89 (40.5)	10 (31.3)	15 (25)	
M stage, n (%)					–
	M0	220 (100)	32 (100)	60 (100)	
	M1	0 (0)	0 (0)	0 (0)	
TNM stage, n (%)					0.000*
	Stage I	73 (33.2)	0 (0)	15 (25)	
	Stage II	61 (27.7)	4 (12.5)	22 (36.7)	
	Stage III	86 (39.1)	28 (87.5)	23 (38.3)	

a SD: standard deviation. *: p value < 0.05.

**Table 2:** Patient characteristics in each cohort (PredictionNet).

Clinical characteristics		Retrospective cohort	TCIA cohort	Prospective cohort	p
Age (years), (mean ± SD)		64.52 ± 10.912	65.53 ± 10.061	63.33 ± 11.613	0.624
Sex, n (%)					0.484
	Male	223 (75.1)	26 (81.3)	42 (70)	
	Female	74 (24.9)	6 (18.8)	18 (30)	
HER2 status, n (%)					0.809
	HER2_ negative	160 (53.9)	16 (50)	30 (50)	
	HER2_ positive	137 (46.1)	16 (50)	30 (50)	

### Model performance

**Figure 3:**
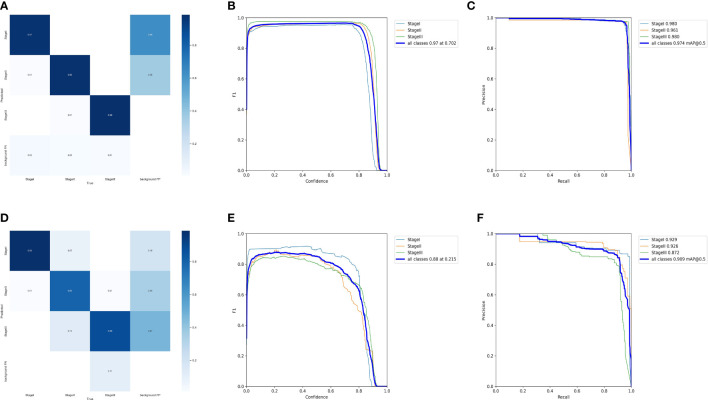
Evaluation of DetectionNet performance in the test and external validation cohort. **(A, B)** The confusion matrix in the test **(A)** and external validation **(B)** cohort. **(B)** The F1 curve. The model had an F1 score of 0.97 in the test cohort. **(C, D)** The P-R curve. The mAP_0.5 value of the test cohort **(C)** and external validation cohort **(D)** were 0.974 and 0.909, respectively. **(E, F)** The F1 curve. The F1 scores of the model in the test cohort **(E)** and external validation cohort **(F)** were 0.97 and 0.88, respectively.

After 150 learning epochs, the Yolov5 achieved the best-optimized parameters, achieving a precision of 0.9717 and a recall of 0.9579 in the test cohort (**Figure S5**). Confusion matrices for the test cohort and external validation cohort were shown in **Figures 3A, B**. The mAP_0.5 value of the test cohort and external validation cohort were 0.974 and 0.909, respectively (**Figures 3C, D**). The F1 scores of the model in the test cohort and external validation cohort were 0.97 and 0.88, respectively (**Figure 3E, F**).

**Figure 4:**
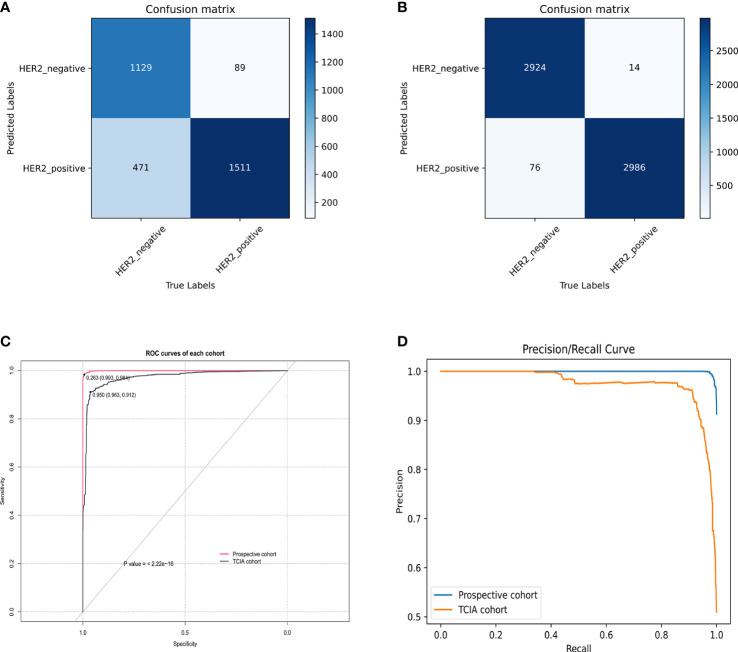
Evaluation of PredictionNet performance. **(A, B)** The confusion matrix in the TCIA **(A)** and prospective **(B)** cohort. **(C)** The ROC curves. The AUC values of TCIA cohort and prospective cohort were 0.9721 and 0.9995, respectively. **(D)** The P-R curves.

**Figure 5:**
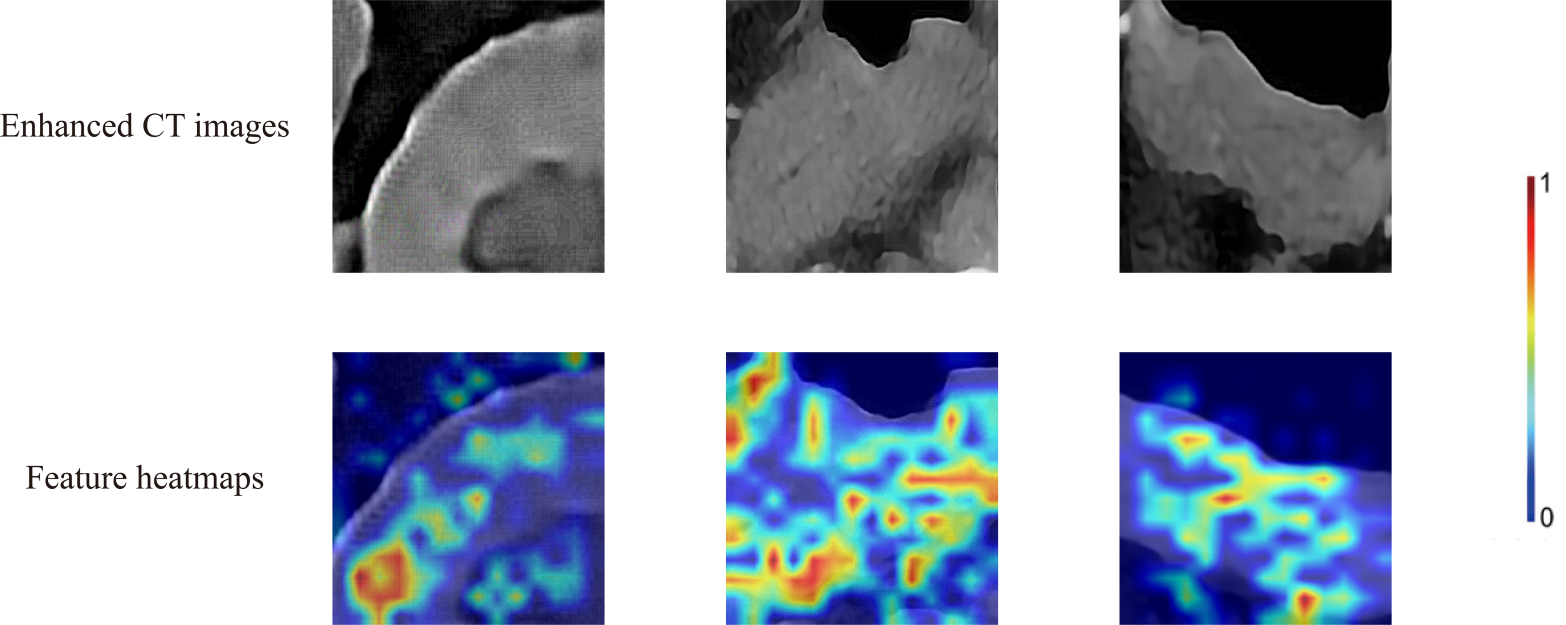
Enhanced CT arterial phase images and feature heatmaps generated by VIT. The importance of features is represented by color bars.

According to the training loss and accuracy value, after 160 learning epochs, all the networks achieved the best-optimized parameters (**Figure S6**). The results showed that the VIT had the best classification results and outperformed CNNs in the test cohort (**Figure S6**). VIT was selected to build the PredictionNet and the confusion matrix showed that the PredictionNet had good classification performance (**Figures 4A, B**). Besides, **Figure 4C** showed excellent performance in the TCIA cohort and prospective cohort with the AUC of 0.9721 and 0.9995, respectively. **Figure 4D** showed the P-R curve of the PredictionNet. **Figure 5** showed representative images of Grad-CAM for the VIT.

### Application of DLS

After training DetectionNet for tumor detection and staging and PredictionNet for HER2 status prediction, we combined these networks to implement the DLS. DLS accepted raw enhanced CT images as input. The system automatically detected the input images, realized tumor detection and staging, and outputted the detection results with image blocks (**Figures S7A, B**). Then, the tumor image was input into the DLS to predict the HER2 status, and the prediction conclusion was output for the doctor to check (**Figure S7C**). In addition, we design a simple web service to be applied to the hospital’s local area network to make the prediction process more accessible to clinicians lacking AI knowledge (**Figure S7D**).

## Discussion

In this study, we developed and validated the enhanced computed tomography-based deep learning system for preoperative prediction of stage and HER2 status in gastric cancer patients. DLS successfully stratified gastric cancer patients according to the stage and HER2 status, facilitating individualized preoperative assessment of stage and HER2 status. More importantly, we built the web service for preoperative prediction of stage and HER2 status in gastric cancer patients.

Accurate and effective stage assessment and HER2 examination play a crucial role in the treatment and prognosis of patients with gastric cancer (4, 5, 15). Medical imaging is a commonly used method for preoperative staging assessment, but the accuracy is not satisfactory (5, 6). Gastroscopic biopsy is a common method for preoperative detection of HER2 status. However, it can lead to serious complications such as infection, bleeding, and perforation (41). Studies have attempted to assess HER2 status through positron emission tomography (PET/CT) and magnetic resonance imaging (MRI) (42, 43). Although certain results have been achieved, they are not routine preoperative examinations for gastric cancer patients. Enhanced CT is more commonly used in the examination and treatment of tumors (31, 44). To our knowledge, this is the first study using enhanced CT images of gastric cancer and deep learning to preoperatively predict the stage and HER2 status.

Different from other studies, most of the deep learning research in the field of gastric cancer focuses on the classification and prognostic analysis of endoscopic images or pathological images (45–48). Compared with endoscopy and tissue biopsy, enhanced CT is a non-invasive preoperative routine test with few risks (49). Furthermore, this study successfully established a DLS and tested the results with the TCIA cohort and the independent prospective cohort. Our findings confirmed that enhanced CT, as a routine preoperative examination in gastric cancer patients, had an inherent feature of receptor expression and thus could reflect the expression status of HER2. Several studies have reported correlations between CT and genes for lung and colorectal cancers (50, 51). The performance of DLS was excellent, achieving the AUC of 0.9721 and 0.9995 in the external validation cohort and prospective cohort, respectively. Due to various reasons like deep learning ‘black box properties’ and clinician bias, DLS is not yet sufficient to replace endoscopic biopsy. However, it was worth noting that DLS had shown advantages over endoscopy and tissue biopsy because it can be assessed from the entire tumor and may be useful if the biopsy was of poor quality. The results of this study underscored the fact that enhanced CT of gastric cancer had inherent features to assess the expression status of HER2 in gastric cancer. It was quite valuable because it was nearly impossible for clinicians to determine the status of HER2 with enhanced CT. Grad-CAM visualized the output of the deep learning models and further research should be carried out based on this result in the future.

Besides, DLS successfully performed tumor detection and preoperative staging prediction on CT images of gastric cancer patients. Previous studies generally only focused on a certain stage such as the depth of tumor invasion or lymph node metastasis and did not conduct an overall assessment (45, 52, 53). Clinical guidelines require clinicians to determine TNM staging before initiating any treatment (54). The study by Huang et al. (55) showed that integrating multiple markers into one model facilitates individualized management of patients and is superior to using a single marker. We were inclined to this view. Only focusing on T staging or N staging may not be able to comprehensively assess the patient’s condition, thus affecting clinicians’ diagnosis, treatment, and prognosis evaluation of patients.

After reviewing the literature, we found that many CT-based deep learning studies use CT layers ranging from one to several dozen layers (33, 52, 56–59). However, we did not review the literature on how many CT layers of input were optimal for deep learning. For clinical work, artificial intelligence needs to process and interpret images quickly and accurately, reducing workflow and medical errors (60). Therefore, it is essential to reduce the workload of doctors while ensuring accuracy. The aim of this study is to build a deep learning system to help clinicians quickly assess patients. If too many CT layers are entered, this increases the workload of the clinician and also increases the hardware configuration conditions required to run the model (61). The study by Hu et al. showed that the performance of building a model with three consecutive CT layers centered on the largest cross-section of the tumor was quite close to that of building a model based on the whole tumor volume (AUC, 0.712 *VS.* 0.725) (62). This provided the basis for our study. Therefore, we screened the five consecutive axial slices with the largest tumor area from the CT images of each patient for the construction of the dataset. The results of our analysis confirmed that both the DetectionNet and the PredictionNet can achieve excellent performance on images based on only five CT layers, which will serve as a reference for other researchers.

Radiomics is an emerging field that has received significant attention in the practice of oncology (31, 63). Li et al. (64) used radiomics to predict the depth of tumor invasion, and Wang et al. (65) predicted lymph node metastasis with an accuracy of 0.77 and 0.80, respectively. Li et al. (66)predicted HER2 status in gastric cancer patients based on CT radiomics, with an AUC of 0.771 in the test cohort. The study by Wang et al. (67)also showed similar results. The accuracy of the DetectionNet and the AUC of the PredictionNet were significantly higher than their radiomics models. The excellent performance of this study may be attributed to the use of deep learning algorithms. The study by Yun et al. (68) showed that integrating deep learning features and radiomics features would reduce the classification performance of deep learning feature models. Chalkidou et al. (69) believed that radiomics features may contain human bias. At the same time, there had always been a problem with reproducibility in radiomics (70). With the advent of deep learning, the value of traditional radiomics has been called into question (71, 72). Deep learning allows relevant features to be learned automatically, without prior definition by the researcher, and these abstract representations also improve learning capabilities, increasing generality and accuracy while reducing potential bias (73). Human-defined radiomics had certain limitations, and the differences between tissue types may not be fully included in the radiomics features.

More importantly, this study also explored the clinical application of deep learning models. Although the previous artificial intelligence research also has excellent performance, they have only been tested in the internal validation cohort or external validation cohort, and have not tried to apply to clinical practice (74, 75), which is not in line with the trend of personalized medicine (76). Schmidt et al. (77) believed that medical research should serve clinical applications. Therefore, we developed the DLS for clinicians. We also built a simple web service for clinicians to use in clinical work. When uploading enhanced CT images of gastric cancer, no professional annotation is required, DLS or web will display brief results of the stage and HER2 prediction. Although there are many difficulties in translating medical research into clinical technology (78), and Cabitza et al. (79) pointed out that artificial intelligence may bring unforeseen consequences in clinical practice, we still believe that this is a worthwhile attempt. Artificial intelligence, with its efficient learning and data processing capabilities, will change the way we deal with gastric cancer and become an invaluable tool for clinicians.

This study has several limitations. First, most of the patients in this study were from a single center, and DLS may not perform well on contrast-enhanced CT images from other hospitals. In our further studies, we will do our best to conduct a multicenter study to reduce the differences between hospitals and make the DLS more robust. In addition, the sample size of the TCIA cohort and prospective cohort of this study was small. Therefore, DLS needs to be validated in larger cohorts. Besides, this study used only enhanced CT arterial phase images for prediction. Another staging of contrast-enhanced CT is for further study. Finally, the DLS of this study was constructed based on the 2D model. In future studies, we will explore the clinical value of 3D models in CT.

In conclusion, our DLS can preoperatively predict the stage and HER2 status in gastric cancer patients based only on enhanced CT images. To make our model more intuitive and convenient for clinicians, we designed a web service based on the developed algorithm. The DLS will help clinicians evaluate the stage and HER2 status of gastric cancer patients preoperatively and select the appropriate treatment, thereby reducing the physical and financial burden on patients.

## Data availability statement

The raw data supporting the conclusions of this article will be made available by the authors, without undue reservation.

## Ethics statement

This study was performed in line with the principles of the Declaration of Helsinki and approved by the Ethics Committee of the Second Affiliated Hospital of Nanjing Medical University (NO [2022].-KY-113-01). The patients/participants provided their written informed consent to participate in this study. Written informed consent was obtained from the individual(s) for the publication of any potentially identifiable images or data included in this article.

## Author contributions

NL collected and organized the clinical data. XG completed the modeling and data analysis and wrote the manuscript. JZ directed the research. All authors contributed to the article and approved the submitted version.

## Funding

This work was supported by the National Natural Science Foundation of China (NO. 81874058).

## Acknowledgments

The authors thanked all colleagues who contributed to this work.

## Conflict of interest

The authors declare that the research was conducted in the absence of any commercial or financial relationships that could be construed as a potential conflict of interest.

## Publisher’s note

All claims expressed in this article are solely those of the authors and do not necessarily represent those of their affiliated organizations, or those of the publisher, the editors and the reviewers. Any product that may be evaluated in this article, or claim that may be made by its manufacturer, is not guaranteed or endorsed by the publisher.
