# Supplementary figures and images for: Accurate preoperative staging and HER2 status prediction of gastric cancer by the deep learning system based on enhanced computed tomography
Source: Front Oncol. 2022 Nov 14;12:950185. doi: 10.3389/fonc.2022.950185 (PMC9702985; doi:10.3389/fonc.2022.950185)

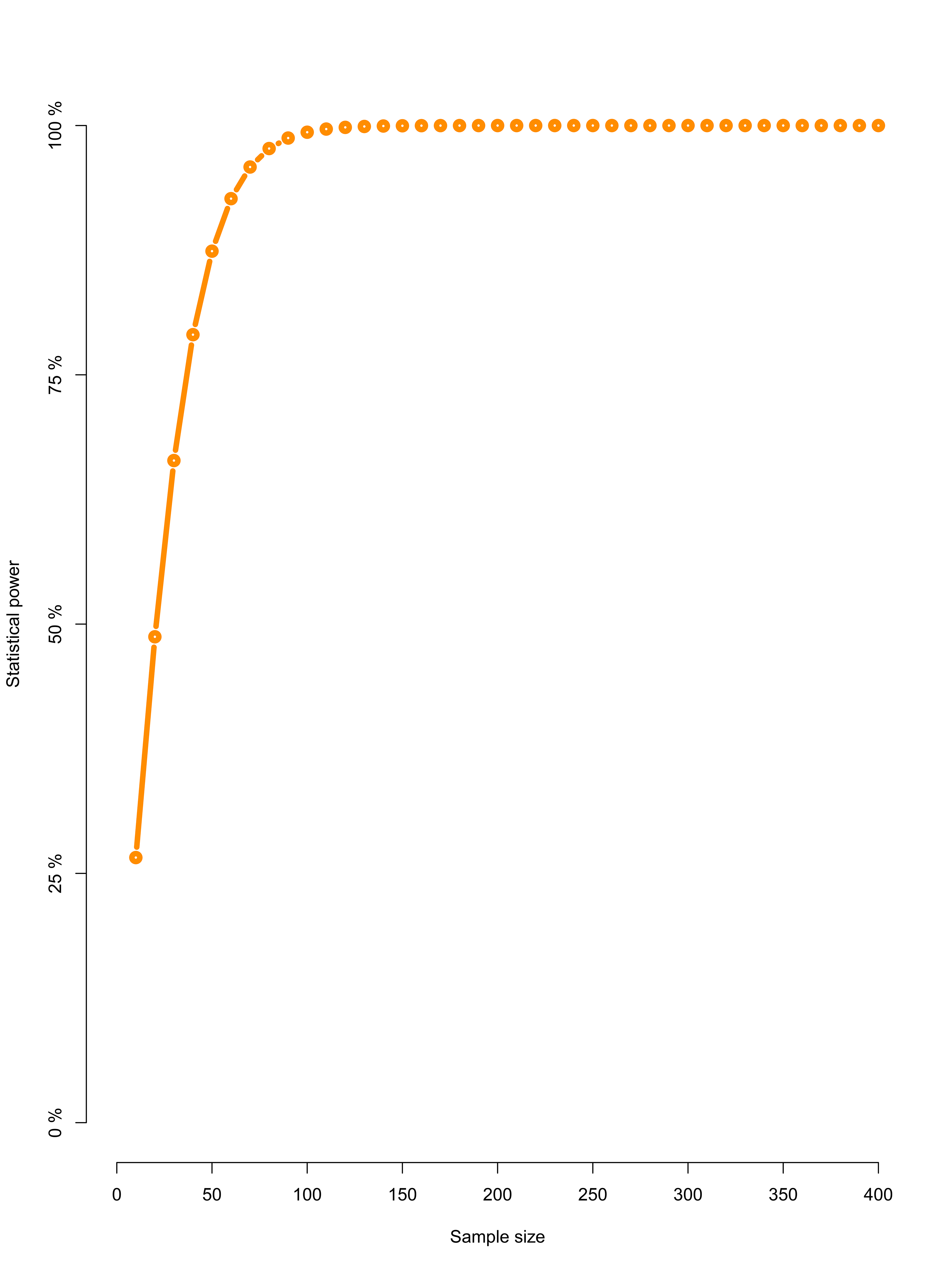

Supplement: Supplementary file 1 [file Image_1.tif]

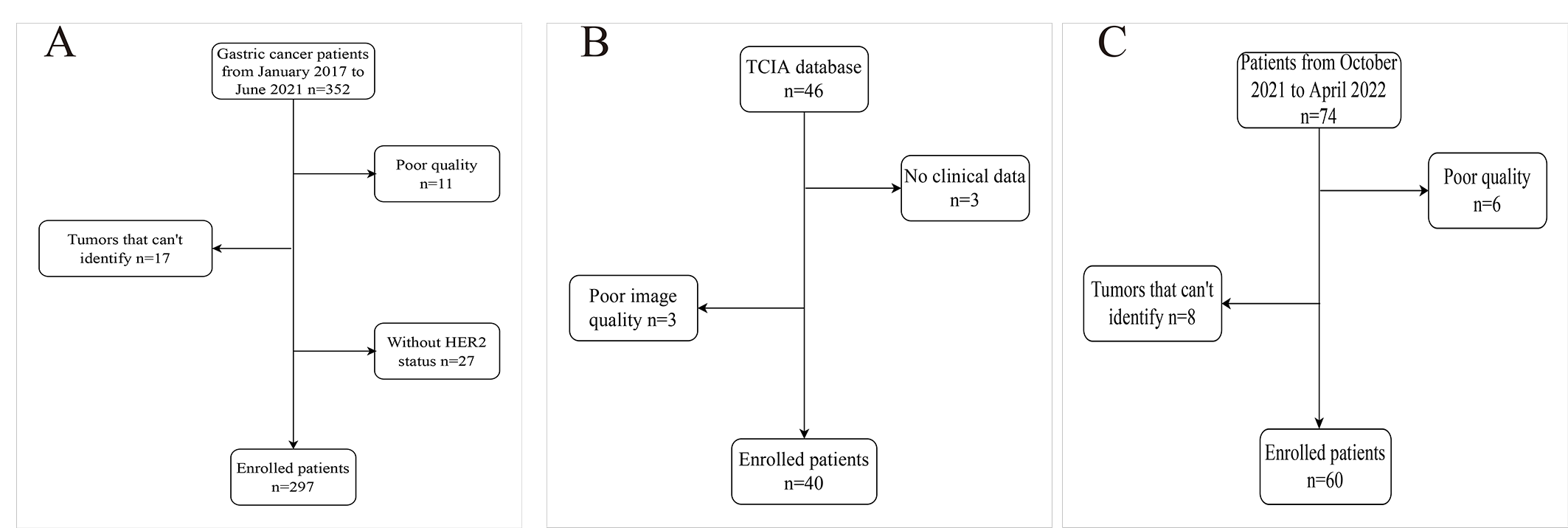

Supplement: Supplementary file 2 [file Image_2.tif]

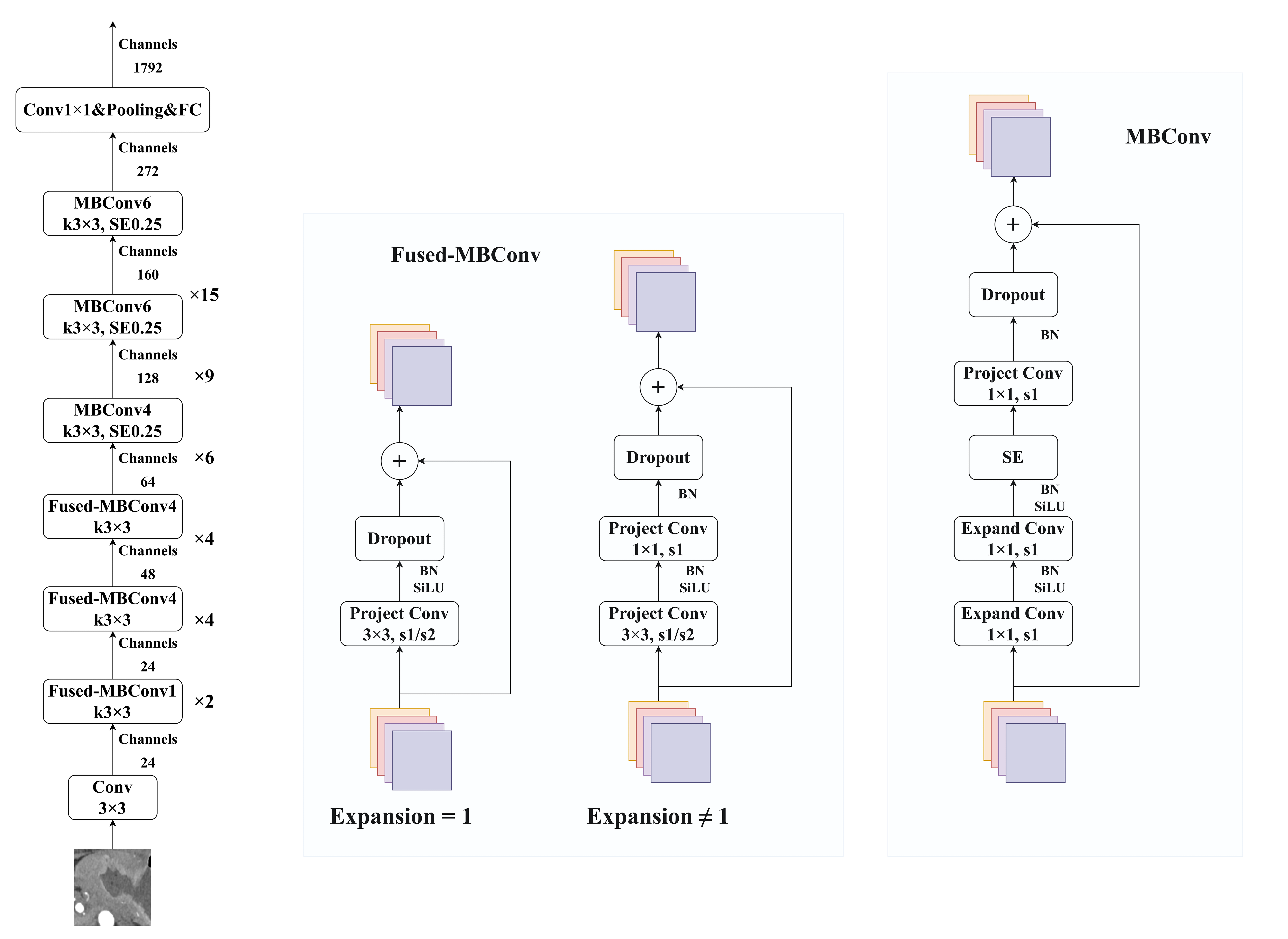

Supplement: Supplementary file 4 [file Image_4.tif]

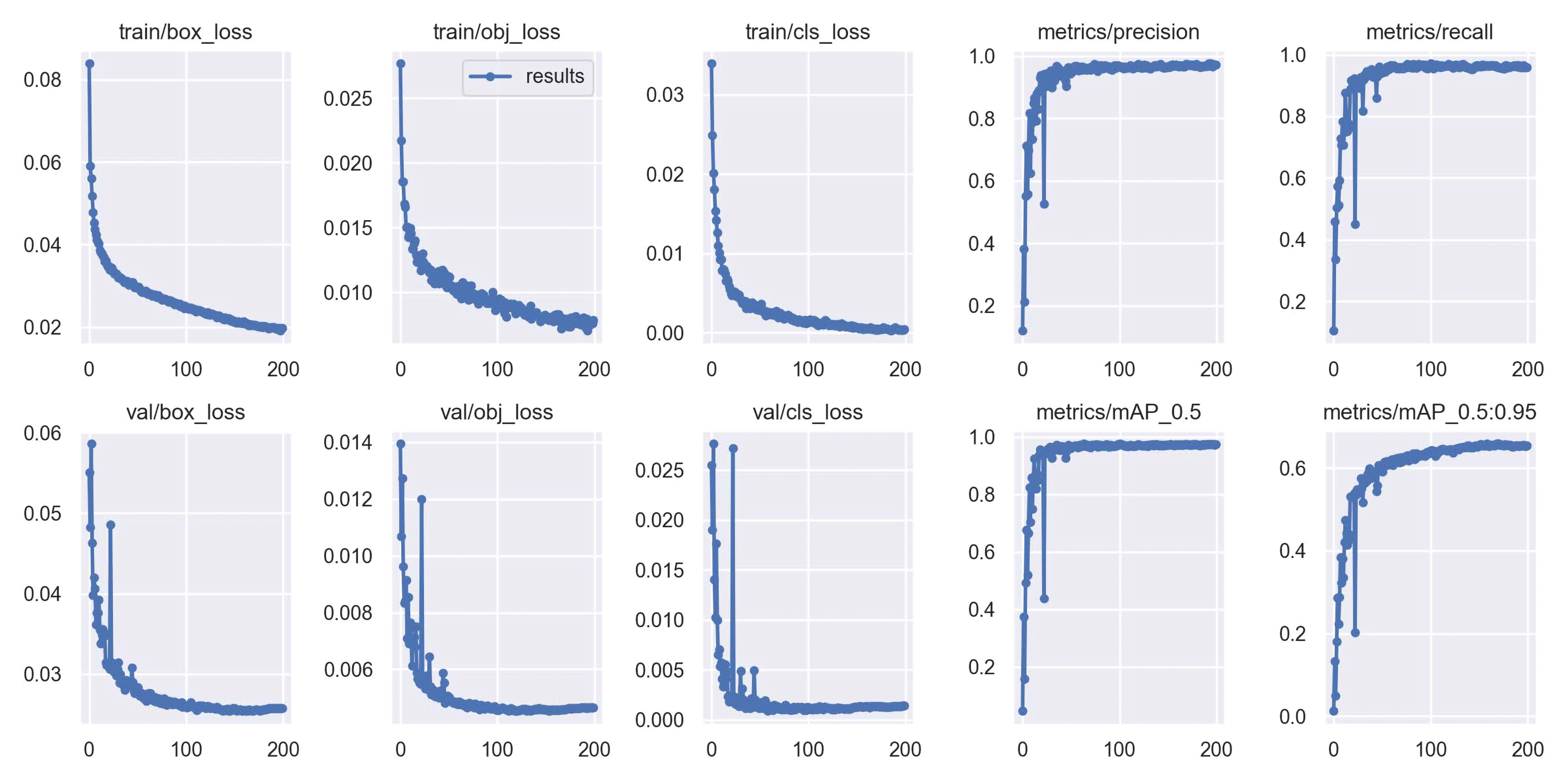

Supplement: Supplementary file 5 [file Image_5.tif]

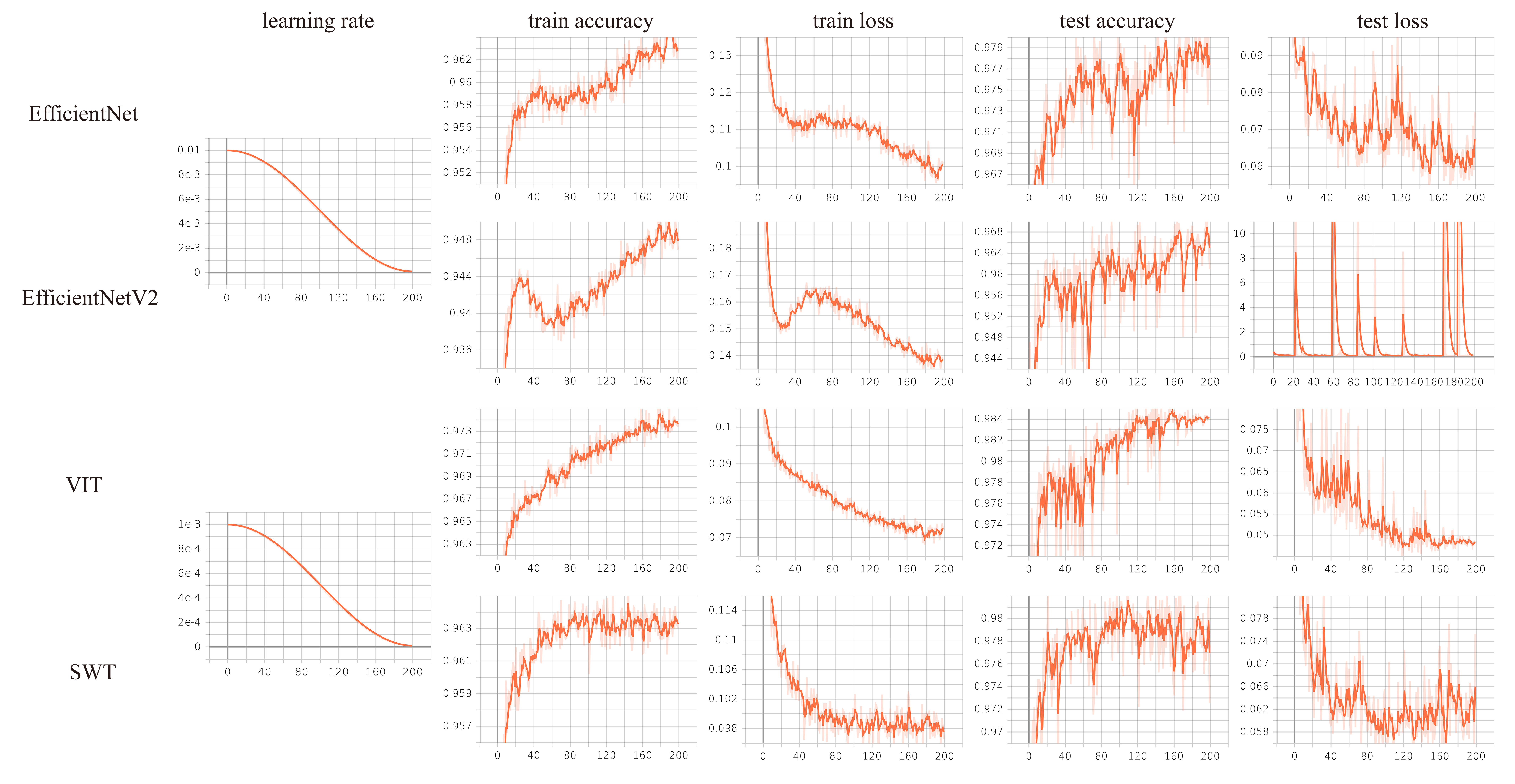

Supplement: Supplementary file 6 [file Image_6.tif]

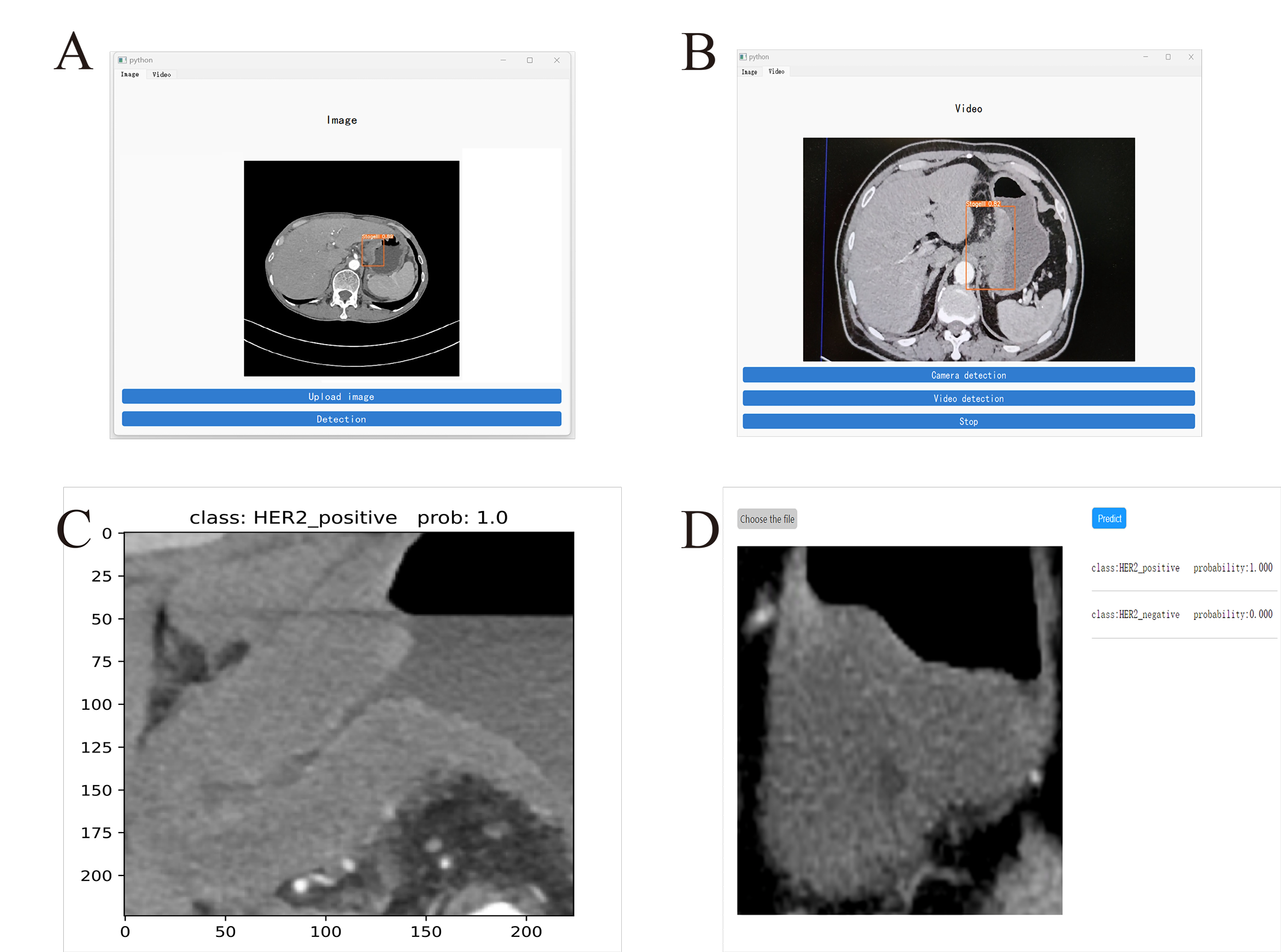

Supplement: Supplementary file 7 [file Image_7.tif]
