# Supplementary material for: Accurate preoperative staging and HER2 status prediction of gastric cancer by the deep learning system based on enhanced computed tomography
Source: Front Oncol. 2022 Nov 14;12:950185. doi: 10.3389/fonc.2022.950185 (PMC9702985; doi:10.3389/fonc.2022.950185)
Supplement: Supplementary file 8 [file DataSheet_1.docx]

Supplementary Material

# Sample Size Assessment

We hypothesized that the deep learning system could improve the prediction accuracy of HER2 status to 90%, given that the reported accuracy was 64.4% for PET/CT [1]. We performed the proportional power calculation of the binomial distribution. The results showed that 79 patients were required to distinguish the classification model from PET/CT with 90% confidence and there will be a maximum error of 5%. When the number of patients was 300, this study had a statistical power of 99.98%.

# HER2 status determination

The HER2 status of gastric cancer patients in the retrospective cohort and prospective cohort of this study was measured using surgical specimens within one week after gastrectomy. According to the gastric cancer scoring system, IHC was used to assess HER2 status. An IHC score of 0 or 1+ indicates HER2 negative status, and 3+ indicates HER2 positive status. Cases with an IHC score of 2+ require further FISH testing to confirm HER2 status. If there is gene amplification, it is judged to be HER2 positive, and if there is no gene amplification, it is judged to be HER2 negative.

For the TCIA dataset, we defined patients with HER2 expression in the top 30% as HER2 positive [2, 3].

# The formula for the loss functionn

Lcls​(***c***p​,***c***gt​)=BCEclssig​(***c***p​,***c***gt​;*w*cls​)

Lbox​(***b***p​,***b***gt​)=1-CIOU=1-[IOU-Distance_2^2^/Distance_C^2^-v^2^/(1-IOU+v)]

Lobj​(*po*​pai,*p*iou​)=BCEobjsig​(*po*​,*p*iou​;*w*obj​)

# Neural network training

## DetectionNet

The network architecture of Yolov5 includes the following aspects: Backbone: New CSP-DarkNet53; Neck: SPPF and New CSP-PAN; Head: YOLOV3 Head. We first imported the pre-training weights for YOLOv5l to initialize the weights of the network. Then, we froze the New CSP-DarkNet53 and fine-tuned the weights of the other layers by learning from our dataset. The test and validation cohorts were used to verify the detection performance of YOLOv5. We set the learning rate to 0.01 and the number of epochs to 200. We adopted Multi-scale training (0.5~1.5x). Assuming that the size of the input image is set to 640 × 640 (https://github.com/ultralytics/yolov5), the size of the training is randomly selected between 0.5 × 640 ∼ 1.5 × 640, and the values are all integer multiples of 32.

We also compared SPPF with Yolov4's SPP module. The results showed that the calculation results of the two are exactly the same, but SPPF is more than twice as fast as the SPP calculation.

## PredictionNet

We first import pre-trained weights to initialize the weights of the network. Then, we fine-tune all the weights by learning from our dataset. We didn't freeze any of the layers. The test cohort, TCIA cohort, and prospective cohort were used to validate the classification performance of the network. The training cohort preprocessing methods included RandomResizedCrop, RandomHorizontalFlip, and Normalization. The test cohort was Resized and Normalized.

# Supplementary Figures

## Figure S1

**Supplementary Figure 1.** Data visualization between statistical power and sample size. When the sample size reaches 150, with the increase of the sample size, the statistical power of this study does not change significantly.

## Figure S2

**Supplementary Figure 2.** Flowchart of data collection for retrospective cohorts (A), TCIA cohorts (B), and prospective cohorts (C).

## Figure S3

**Supplementary Figure 3.** The overall network structure of the EfficientNet.

## Figure S4

**Supplementary Figure 4.** The overall network structure of the EfficientNetV2.

## Figure S5

**Supplementary Figure 5.** The variation of each metric in Yolov5 over 200 epochs. mAP_0.5: mean Average Precision (IoU=0.5). mAP_0.5:0.95: represent the average mAP at different IoU thresholds (from 0.5 to 0.95 in steps of 0.05) (0.5, 0.55, 0.6, 0.65, 0.7, 0.75, 0.8, 0.85, 0.9, 0.95). After 150 learning epochs, the Yolov5 achieved the best-optimized parameters, achieving a precision of 0.9717 and a recall of 0.9579 in the test cohort.

## Figure S6

**Supplementary Figure 6.** Variation of each metric over 200 epochs for different networks. According to the training loss and accuracy value, after 160 learning epochs, all the networks achieved the best-optimized parameters. The VIT model had the best classification results and outperformed CNNs in the test cohort.

## Figure S7

**Supplementary Figure 7.** Illustration for DLS and the web. (A): DLS detects tumor location and stage prediction on enhanced CT image. (B): DLS detects tumor location and stage prediction on enhanced CT video. (C): DLS predicts HER2 status. (D): Web service for predicting HER2 status.

# Reference

1. Chen, R.; Zhou, X.; Liu, J.; Huang, G., Relationship Between 18F-FDG PET/CT Findings and HER2 Expression in Gastric Cancer. Journal of nuclear medicine : official publication, Society of Nuclear Medicine 2016, 57 (7), 1040-4.

2. Boku, N., HER2-positive gastric cancer. Gastric cancer : official journal of the International Gastric Cancer Association and the Japanese Gastric Cancer Association 2014, 17 (1), 1-12.

3. Lordick, F.; Al-Batran, S. E.; Dietel, M.; Gaiser, T.; Hofheinz, R. D.; Kirchner, T.; et al., HER2 testing in gastric cancer: results of a German expert meeting. Journal of cancer research and clinical oncology 2017, 143 (5), 835-841.
